# The Journey of an Erupting Permanent Tooth and Its Fibrous Companion: A Pediatric Case Series

**DOI:** 10.7759/cureus.104211

**Published:** 2026-02-25

**Authors:** Mridula Goswami, Riya Marie Johnson, Babita Jangra, Vishal Vishal, Manisha Manisha

**Affiliations:** 1 Department of Pediatric and Preventive Dentistry, Maulana Azad Institute of Dental Sciences, New Delhi, IND

**Keywords:** fibrous hyperplasia, laser therapy, mechanical trauma, pediatric and preventive dentistry, reactive hyperplastic lesions

## Abstract

Reactive proliferative lesions are commonly encountered in the oral cavity, primarily attributable to the continuous exposure of the mucosal tissues to recurrent mechanical trauma and microbial challenges. These lesions typically arise secondary to identifiable initiating factors, including dental plaque accumulation, calculus deposition, persistent local irritation, or the presence of foreign bodies. Reactive hyperplastic lesions represent non-neoplastic enlargements of the oral soft tissues that develop as an amplified reparative response following chronic irritation or minor trauma. The present case series documents an uncommon presentation of fibrous hyperplasia in pediatric patients, occurring in association with the eruption of permanent teeth. This report emphasizes the importance of recognizing that physiological dental processes, such as tooth eruption, may act as contributory factors in the genesis of reactive lesions. Furthermore, it highlights the critical role of histopathological assessment in establishing a definitive diagnosis. Early intervention of these lesions is crucial, as longstanding lesions in the presence of chronic irritation have the potential to transform into neoplasms.

## Introduction

The oral cavity plays an essential role in several fundamental functions, such as speech, mastication, and respiration. In children, proper development and maintenance of oral tissues are crucial for overall health and well-being. However, the oral mucosa is frequently exposed to various mechanical, chemical, and biological irritants, making it susceptible to the development of reactive lesions. Among these, reactive hyperplastic lesions (RHLs) represent a significant group of oral soft tissue lesions that arise as a result of chronic irritation or trauma to the oral mucosa [1]. These lesions are benign in nature and represent an exaggerated reparative response rather than true neoplastic proliferation. They are characterized histologically by the proliferation of fibroblasts, inflammatory cells, and vascular components, resulting in localized tissue overgrowth [2].

RHLs constitute approximately 5-15% of all oral lesions and are commonly encountered in both pediatric and adult populations. Based on their clinical and histopathological characteristics, they are classified into irritation fibroma (fibrous hyperplasia), pyogenic granuloma, peripheral ossifying fibroma, and peripheral giant cell granuloma [3,4]. The gingiva is the most frequently affected site, accounting for approximately 57.4% of cases, followed by the buccal mucosa (28.4%), due to its increased susceptibility to chronic irritation and trauma [4].

The prevalence of RHLs varies with age. In pediatric patients, pyogenic granuloma is the most common RHL, accounting for approximately 30-40% of cases, whereas fibrous hyperplasia represents around 15-25%. In contrast, in adults, fibrous hyperplasia is the most prevalent lesion, comprising approximately 35-45% of cases, followed by pyogenic granuloma (25-30%), peripheral ossifying fibroma (10-20%), and peripheral giant cell granuloma (5-10%) [3,4].

These lesions typically arise due to chronic local irritation or trauma, most commonly associated with dental plaque and calculus resulting from poor oral hygiene (60-70%), followed by mechanical trauma from sharp tooth margins (10-20%), irritation from ill-fitting dental appliances or restorations (5-15%), and parafunctional habits such as lip or cheek biting (5-10%). Hormonal influences and nutritional deficiencies, such as vitamin C and iron deficiency, may further exacerbate tissue response. Since children are more susceptible to dental trauma and developing oral habits, they are at an increased risk of developing RHLs [3].

A retrospective study by Sangle et al. (2018) reported that RHLs accounted for approximately 11.7% of all oral mucosal pathologies, with a higher prevalence observed in females and peak incidence in the second and third decades of life. Clinically, traumatic fibroma was the most frequently diagnosed lesion (37.4%), while fibrous hyperplasia was the most common histopathological finding (37.4%) [5].

Fibrous hyperplasia, also referred to as irritation fibroma or focal fibrous hyperplasia, is a common subtype of RHL, characterized by localized proliferation of dense fibrous connective tissue in response to persistent local irritation. It represents a reactive, non-neoplastic process and commonly occurs in areas subjected to repeated trauma or irritation [6].

This case series describes three cases of fibrous hyperplasia in pediatric patients occurring in association with erupting permanent teeth, highlighting the role of eruption-related irritation as a potential etiological factor.

## Case presentation

This case series describes the occurrence of fibrous hyperplasia in three pediatric patients aged between eight and 13 years, associated with the eruption of permanent teeth. All patients presented to the department of pediatric and preventive dentistry with distinct chief complaints. Informed parental consent and child assent were obtained prior to treatment. Each patient underwent a comprehensive evaluation, including detailed medical and dental history, radiographic assessment, and thorough intraoral clinical examination. All cases demonstrated an inadequate oral hygiene status.

Case 1

An 11-year-old female patient presented with a chief complaint of pain and swelling in the right mandibular posterior region for the past two weeks. The parent reported a history of extraction of the primary second molar (85) due to discomfort. As per the parent, one day following extraction, the patient developed mild redness and swelling at the affected site, which gradually increased in size, resulting in pain and difficulty during mastication.

**Figure 1:**
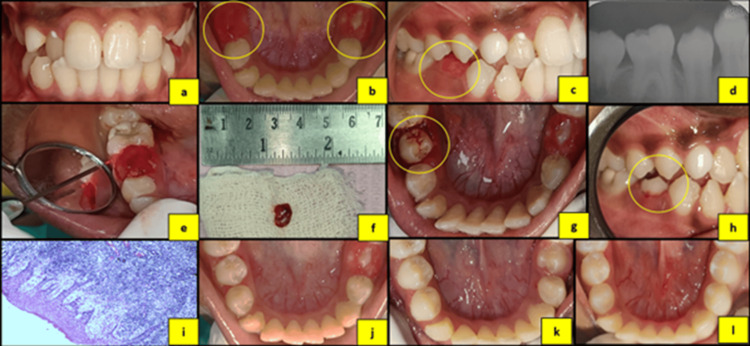
(a) Preoperative frontal view. (b) Preoperative mandibular occlusal view. (c) Preoperative right lateral view showing impingement of the opposing premolar on the developing lesion. (d) Intraoral periapical radiograph (IOPA) of tooth #35. (e) Laser excision of the lesion. (f) Excised tissue specimen. (g) Immediate postoperative occlusal view. (h) Immediate postoperative lateral view. (i) Histopathological image of the specimen. (j) One-day postoperative view. (k) Two-week postoperative view. (l) Four-week postoperative view.

On intraoral examination, a dome-shaped, sessile, erythematous growth measuring approximately 6 × 4 mm was observed on the alveolar ridge, extending from the mesial surface of tooth 46 to the distal surface of tooth 44. The lesion was soft and edematous in consistency with well-defined borders (Figure 1A). The maxillary right second premolar (15) was observed to be impinging on the lesion.

An intraoral periapical radiograph (IOPA) revealed the underlying tooth bud of the erupting mandibular right second premolar (45) corresponding to Nolla’s stage 8, with no associated pathological changes.

Additionally, the contralateral mandibular left second premolar (35) exhibited erythematous and inflamed gingival tissues suggestive of eruption-related gingivitis (Figure 1). Symptomatic management was instituted, including topical analgesics, warm saline rinses, and 0.2% (w/v) chlorhexidine mouthwash.

Case 2

A 13-year-old female patient presented with a chief complaint of swelling of the palatal area to the upper left central incisor since its eruption, which had gradually increased in size and was associated with discomfort during mastication.

**Figure 2:**
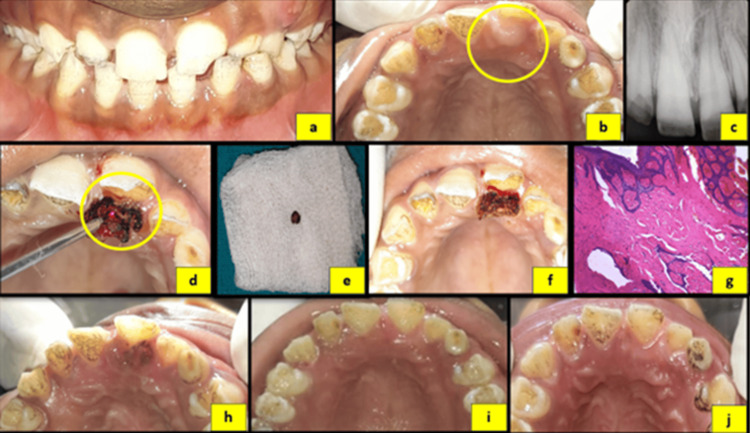
(a) Preoperative frontal view. (b) Preoperative maxillary occlusal view. (c) Intraoral periapical radiograph (IOPA) of tooth #21. (d) Laser excision of the lesion. (e) Excised tissue specimen. (f) Immediate postoperative occlusal view. (g) Histopathological images of the specimen. (h) One-day postoperative view. (i) Two-week postoperative view. (j) Four-week postoperative view.

On intraoral examination, a well-defined, dome-shaped, sessile growth measuring approximately 5 × 4 mm was observed on the palatal aspect of tooth 21. The lesion was firm in consistency, covered by normal-appearing mucosa, and exhibited mild tenderness on palpation. The patient also reported impingement of the mandibular incisors on the swelling during normal occlusion. Additionally, tooth 21 was noted to be out of arch alignment and exhibited an Ellis class II fracture, with a history of trauma two months prior (Figure 2).

An IOPA revealed no associated pathological changes. The tooth was asymptomatic and demonstrated a normal response to pulp vitality testing.

Case 3

An eight-year-old female patient presented with a chief complaint of swelling of the palatal area in the upper right front tooth region for the past one month. The patient had no significant previous dental history.

**Figure 3:**
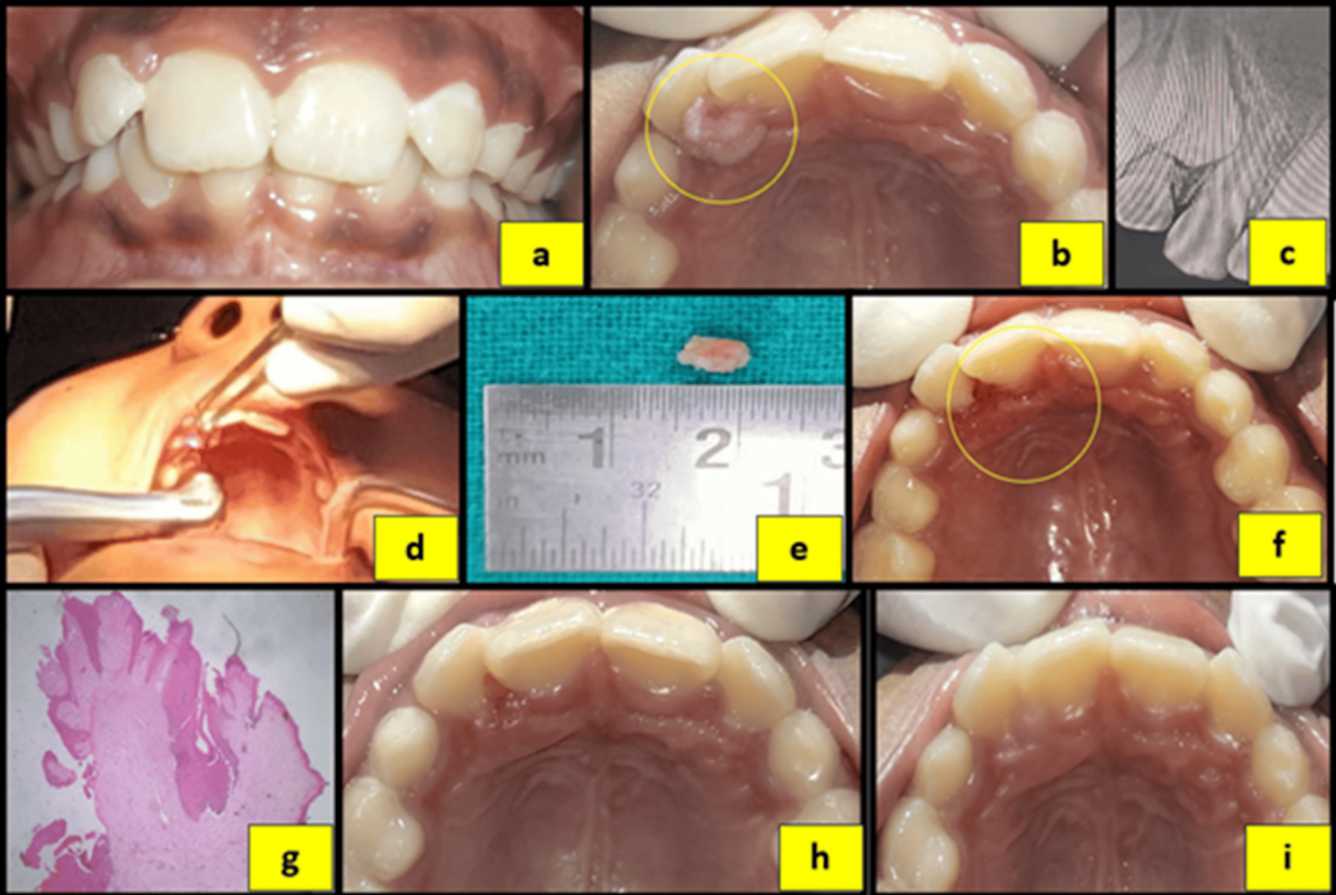
(a) Preoperative frontal view showing an anterior deep bite. (b) Preoperative maxillary occlusal view. (c) Intraoral periapical radiograph (IOPA) of tooth #12. (d) Laser excision of the lesion. (e) Excised tissue specimen. (f) Immediate postoperative occlusal view. (g) Histopathological images of the specimen. (h) One-day postoperative view. (i) Four-week postoperative view.

On intraoral examination, a dome-shaped, sessile growth measuring approximately 4 × 5 mm was observed on the palatal aspect of the erupting maxillary right lateral incisor (12). The lesion was firm in consistency, non-tender on palpation, had well-defined borders, and was covered by pinkish-white mucosa. Additionally, the patient exhibited a deep bite, which resulted in impingement of the mandibular incisors onto the swelling (Figure 3).

An IOPA revealed the underlying tooth bud of the erupting tooth 12 corresponding to Nolla’s stage 8, with no associated pathological changes.

Treatment and follow-up

Based on clinical and radiographic assessment, a provisional diagnosis of pyogenic granuloma was made in all three cases. Surgical excision of the lesions was performed using a diode laser (Primo Dental Diode Laser, Medency, Vicenza, Italy) at 980 nm, 2.4 W, and 100 Hz in two cases, and an Er,Cr:YSGG laser (Waterlase iPlus, Biolase, Foothill Ranch, CA) at 1.0-1.8 W and 10-15 Hz in the third case, in accordance with the manufacturer’s guidelines. The excised specimens were submitted for histopathological examination. Histopathological analysis revealed dense collagenous connective tissue containing numerous spindle-shaped fibroblasts, blood vessels of varying calibers, and an overlying hyperplastic stratified squamous epithelium, confirming the diagnosis of fibrous hyperplasia (Figures 1I, 2G, 3G).

Postoperative instructions regarding maintenance of oral hygiene were provided to all patients. Healing was uneventful, with no postoperative complications or recurrence observed. The patients were kept under periodic follow-up. Successful eruption of the associated permanent teeth and no recurrence of the lesion was observed during subsequent follow-up visits in all cases (Figures 1G, 2F, 3F). The patients are being monitored regularly to evaluate long-term outcomes and to detect any future recurrence.

## Discussion

In pediatric patients, RHLs are commonly associated with chronic local irritants such as poor oral hygiene, lip or cheek biting, or irritation from orthodontic appliances. Although reactive gingival lesions associated with natal and neonatal teeth have been reported in the literature, their occurrence remains relatively uncommon. Sethi et al. (2015) and Singh et al. (2004) reported cases of fibrous hyperplasia associated with natal teeth, while Singh et al. (2021) described a case of peripheral ossifying fibroma associated with a neonatal tooth [7-9].

In the present case series, the development of fibrous hyperplasia may be attributed to chronic mechanical irritation from erupting permanent teeth in conjunction with inadequate oral hygiene. The process of tooth eruption is associated with localized inflammation and remodeling of the surrounding soft tissues, which may stimulate an exaggerated fibroblastic reparative response. Furthermore, repeated trauma from impingement by opposing dentition may act as an additional source of irritation, contributing to persistent tissue injury and subsequent reactive fibrous tissue proliferation [10,11].

This case series documents fibrous hyperplasia occurring in association with the eruption of permanent teeth in pediatric patients. The condition typically presents as a localized, soft, and painless mucosal overgrowth on the gingiva, buccal mucosa, or other regions subjected to repetitive irritation. Although generally asymptomatic, these lesions may occasionally cause discomfort during mastication or oral hygiene practices [12].

The clinical presentation of fibrous hyperplasia closely resembles that of other RHLs as well as certain benign neoplastic proliferations, thereby posing a significant diagnostic challenge. Differentiation based solely on clinical features is often difficult, necessitating a comprehensive differential diagnosis when evaluating gingival overgrowths. The various lesions considered in the differential diagnosis are summarized in Table 1 [5,12]. In the present cases, the lesions presented as localized gingival overgrowths in association with erupting permanent teeth and exhibited clinical features such as localized enlargement, erythematous appearance, and soft to firm consistency, along with signs of chronic irritation. These clinical findings were suggestive of pyogenic granuloma, which is a commonly encountered reactive lesion in the pediatric population.

**Table 1:** Features of various lesions considered in the differential diagnosis. Reference [5,12].

S. No.	Lesion	Clinical features	Radiographic features	Histopathological findings
1.	Pyogenic granuloma	Red, soft, ulcerated mass, prone to bleeding; typical rapid growth seen on the gingiva and tongue	No radiographic changes	Proliferative capillary network within a loose connective tissue stroma; mixed inflammatory infiltrate of neutrophils and lymphocytes
2.	Peripheral ossifying fibroma	Nodular mass on the gingiva, firm to palpation, often pink/red with calcified foci	Alveolar bone resorption or calcifications	Fibrous stroma with calcified material, bone, or cementum; osteoblastic rimming
3.	Peripheral giant cell granuloma	Purplish or reddish mass on the gingiva, often soft and compressible; may ulcerate and bleed easily	Cupping resorption of the underlying alveolar bone	Multinucleated giant cells within a vascular stroma; hemosiderin deposits and chronic inflammatory cells
4.	Fibrous hyperplasia	Firm, smooth, asymptomatic, usually <1 cm; common on the buccal mucosa, tongue, and gingiva	No radiographic changes	Dense collagenized stroma with few cells and occasional inflammation; covered by normal or keratinized epithelium
5.	Drug-induced gingival overgrowth	Diffuse gingival enlargement; may be localized to the anterior gingiva or generalized; firm and non-painful	No radiographic changes	Dense collagen fibers with increased cellularity, reduced inflammation; occasionally fibrosis with epithelial hyperplasia
6.	Fibrous epulis	Firm, painless, pink/red swelling on the gingiva, typically near the interdental papillae	No radiographic changes	Fibrous tissue with fibroblasts and collagen bundles; often a few inflammatory cells

However, owing to the significant overlap in clinical features between pyogenic granuloma and fibrous hyperplasia, a definitive diagnosis could not be established based on clinical examination alone. Histopathological examination of the excised specimen subsequently confirmed the diagnosis of fibrous hyperplasia. This underscores the critical importance of histopathological evaluation as the gold standard for definitive diagnosis and for accurate differentiation from other clinically similar reactive and neoplastic lesions [13].

The recommended management for such lesions is complete surgical excision, which may be performed using a conventional scalpel, laser systems, or electrocautery. In a double-blinded comparative study, de Jesus et al. (2020) evaluated diode laser and electrocautery for the removal of inflammatory fibrous hyperplasia and concluded that diode laser therapy demonstrates equivalent efficacy and safety to electrocautery when used under comparable clinical conditions [14]. Similarly, Noble et al. (2019), in a case series assessing three therapeutic approaches for irritational fibroma, reported that both laser and electrocautery techniques offer superior hemostasis compared with scalpel excision. Additionally, laser excision provides further advantages, including minimal intraoperative bleeding, reduced postoperative pain, decreased risk of infection, and enhanced healing, and provides precise tissue removal with minimal damage to adjacent tissues, which is especially beneficial in pediatric patients where cooperation and comfort are critical [15]. Here, different laser systems were used based on equipment availability, and both demonstrated effective and safe outcomes.

Early management is essential to avoid interference with oral functions and ensure good healing. Fibrous hyperplasia has a low recurrence rate, ranging from approximately 0% to 8%, particularly when complete surgical excision and elimination of local irritational factors are achieved. Recurrence is usually associated with incomplete removal of the lesion or persistence of chronic irritation [4,6]. In the present cases, the patients were monitored periodically and are currently under continuous follow-up. No recurrence has been observed to date. However, long-term follow-up is recommended to ensure complete healing and to facilitate early detection of any potential recurrence.

This case series has certain limitations, including a small sample size and limited follow-up duration. Additionally, the exact etiological mechanism cannot be definitively established, and larger longitudinal studies are required to better understand the association between tooth eruption and fibrous hyperplasia. Despite these limitations, the present case series highlights the importance of early diagnosis, histopathological confirmation, and appropriate management.

## Conclusions

The oral cavity is an ideal niche for the manifestation of various reactive overgrowths of soft tissue, which pose a diagnostic dilemma due to their similar clinical presentations. Treatment should be aimed at managing the source of the irritation, as these lesions occur due to continuous tissue trauma and irritation. As the definitive management is surgical excision, lasers can be considered as a good modality even for very large lesions that are difficult to access by conventional surgery. Although these hyperplastic lesions are generally self-limiting in nature, surgical removal becomes necessary when they compromise oral form or function.
